# 2-Bromo-1-phenyl­ethanone

**DOI:** 10.1107/S1600536811014644

**Published:** 2011-04-22

**Authors:** Richard Betz, Cedric McCleland, Harold Marchand

**Affiliations:** a Nelson Mandela Metropolitan University, Summerstrand Campus, Department of Chemistry, University Way, Summerstrand, PO Box 77000, Port Elizabeth 6031, South Africa

## Abstract

The title compound, C_8_H_7_BrO, is a halogenated derivative of acetophenone. The mol­ecule shows noncrystallographic *C_s_* symmetry. The intra­cyclic C—C—C angles cover the range 118.8 (2)–120.4 (3)°. In the crystal structure, C—H⋯O contacts connect the mol­ecules into undulating sheets perpendicular to the crystallographic *c* axis.

## Related literature

For the crystal structure of α-chloro-acetophenone, see: Barrans & Maisseu (1966 ▶); Grossert *et al.* (1984 ▶). For the crystal structure of α-iodo-acetophenone, see: Lere-Porte *et al.* (1982 ▶). For the crystal structures of coordination compounds using the title compound as a ligand, see: Laube *et al.* (1991 ▶). For details of graph-set analysis of hydrogen bonds, see: Etter *et al.* (1990 ▶); Bernstein *et al.* (1995 ▶).
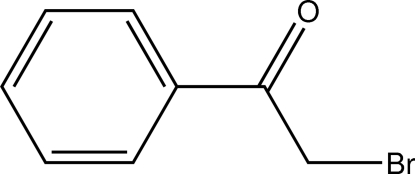


## Experimental

### 

#### Crystal data

C_8_H_7_BrO
*M* *_r_* = 199.05
Orthorhombic, 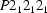

*a* = 4.1459 (2) Å
*b* = 9.6731 (5) Å
*c* = 18.8178 (9) Å
*V* = 754.66 (6) Å^3^
*Z* = 4
Mo *K*α radiation
μ = 5.37 mm^−1^
*T* = 200 K
0.54 × 0.43 × 0.09 mm

#### Data collection

Bruker APEXII CCD diffractometer
Absorption correction: multi-scan (*SADABS*; Bruker, 2010 ▶) *T*_min_ = 0.588, *T*_max_ = 1.000
7436 measured reflections
1867 independent reflections
1692 reflections with *I* > 2σ(*I*)
*R*_int_ = 0.037

#### Refinement

*R*[*F* ^2^ > 2σ(*F* ^2^)] = 0.029
*wR*(*F* ^2^) = 0.072
*S* = 1.08
1867 reflections
91 parameters
H-atom parameters constrained
Δρ_max_ = 0.42 e Å^−3^
Δρ_min_ = −0.66 e Å^−3^
Absolute structure: Flack (1983 ▶), with 736 Friedel pairs
Flack parameter: 0.015 (14)

### 

Data collection: *APEX2* (Bruker, 2010 ▶); cell refinement: *SAINT* (Bruker, 2010 ▶); data reduction: *SAINT*; program(s) used to solve structure: *SHELXS97* (Sheldrick, 2008 ▶); program(s) used to refine structure: *SHELXL97* (Sheldrick, 2008 ▶); molecular graphics: *ORTEP-3* (Farrugia, 1997 ▶) and *Mercury* (Macrae *et al.*, 2008 ▶); software used to prepare material for publication: *SHELXL97* and *PLATON* (Spek, 2009 ▶).

## Supplementary Material

Crystal structure: contains datablocks I, global. DOI: 10.1107/S1600536811014644/fy2008sup1.cif

Supplementary material file. DOI: 10.1107/S1600536811014644/fy2008Isup2.cdx

Structure factors: contains datablocks I. DOI: 10.1107/S1600536811014644/fy2008Isup3.hkl

Additional supplementary materials:  crystallographic information; 3D view; checkCIF report

## Figures and Tables

**Table 1:** Hydrogen-bond geometry (Å, °)

*D*—H⋯*A*	*D*—H	H⋯*A*	*D*⋯*A*	*D*—H⋯*A*
C2—H21⋯O1^i^	0.99	2.46	3.317 (4)	145
C2—H22⋯O1^ii^	0.99	2.44	3.268 (4)	141
C8—H8⋯O1^i^	0.95	2.60	3.442 (3)	148

Symmetry codes: (i) 
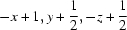
; (ii) 
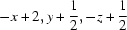
.

**Fig. 1.**
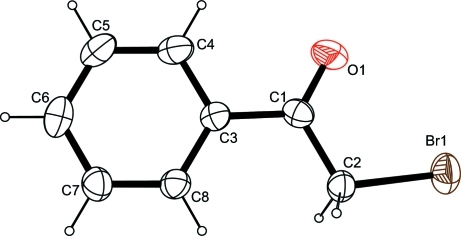
The molecular structure of the title compound, with atom labels and anisotropic displacement ellipsoids (drawn at 50% probability level).

**Fig. 2.**
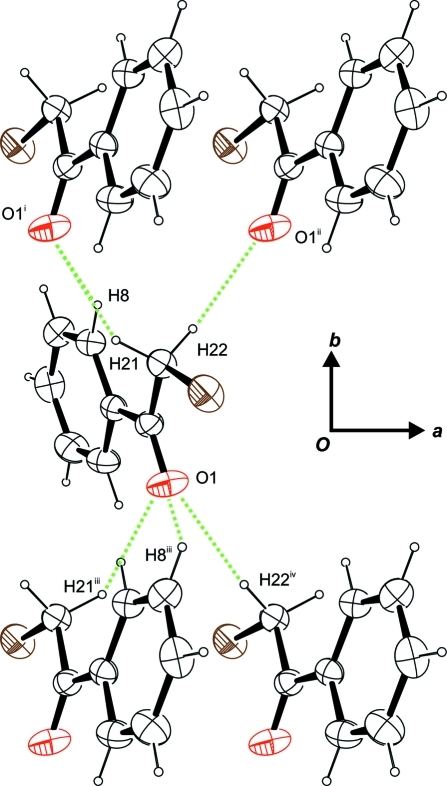
Intermolecular contacts, viewed along [00\=1]. Symmetry operators: i -x + 1, y + 1/2, -z + 1/2; ii -x + 2, y + 1/2, -z + 1/2; iii -x + 1, y - 1/2, -z + 1/2; iv -x + 2, y - 1/2, -z + 1/2.

**Fig. 3.**
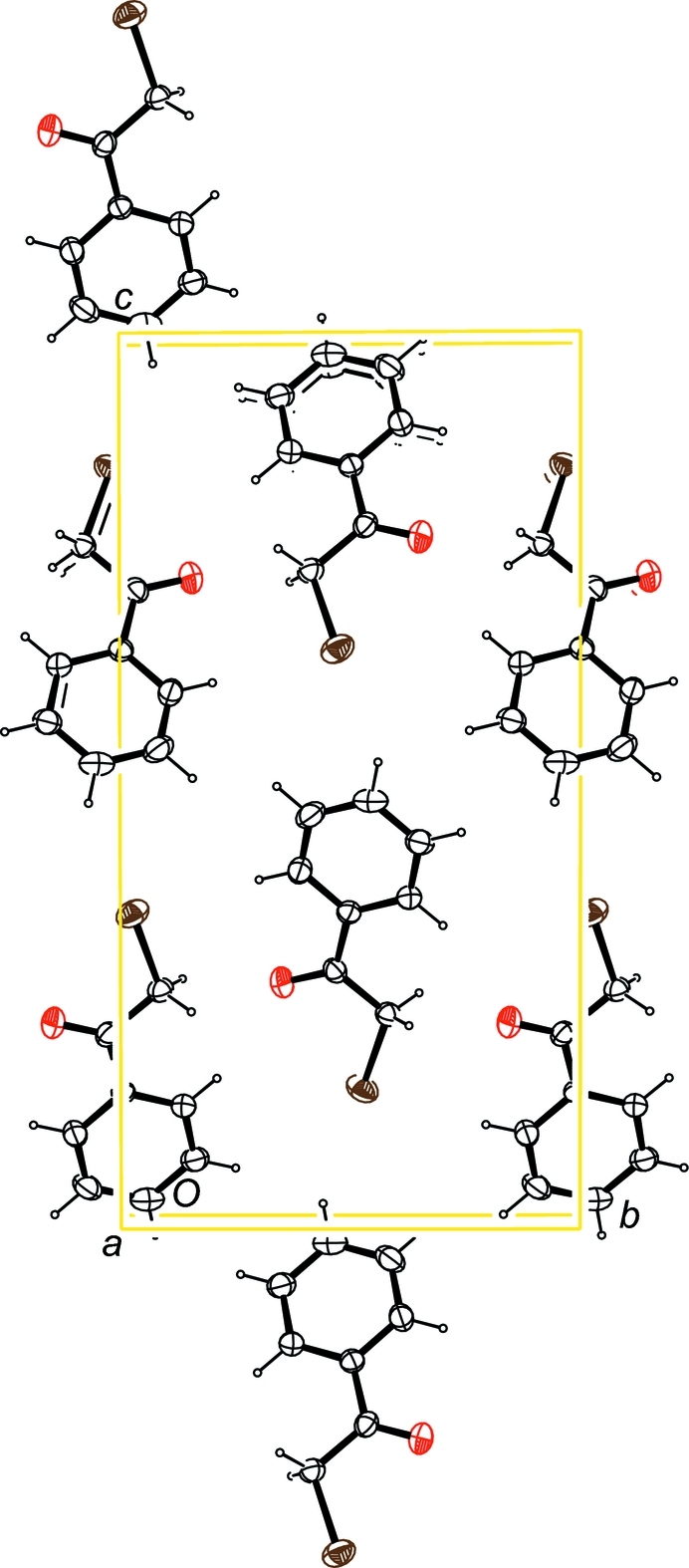
Molecular packing of the title compound, viewed along [\=100] (anisotropic displacement ellipsoids drawn at 50% probability level).

## supplementary crystallographic information

### Comment

Derivatives of acetophenone are widely used in preparative organic chemistry. At the beginning of a comprehensive study about the effects of various substituents on benzo-annulated seven-membered ring systems, the molecular structure of the title compound was determined to enable comparisons with acetophenone-derived target compounds.

Intracyclic C—C—C angles span a range from 118–120°. The smallest angle is found on the C atom bearing the carbonylic substituent while the second smallest one is found on the C atom in *para*-position. The atoms of the aliphatic substituent are nearly coplanar with the aromatic system and its conjugated carbonyl group, the least-squares planes defined by their respective atoms intersect at an angle of only 4.18 (15)°.

In the crystal structure, C—H···O contacts can be observed which stem from both H atoms of the methylene group as well as one of the H atoms in *ortho*-position to the substituent on the phenyl ring. The carbonylic O atom serves as threefold acceptor (Fig. 2). Describing these contacts in terms of graph-set analysis necessitates a *C*(4)*C*(4)*C*(5) descriptor on the unitary level. In total, the molecules are connected to waved sheets perpendicular to the crystallographic *c* axis. The shortest distance between the centroids of two π-systems was measured at 5.8289 (17) Å.

The packing of the compound in the crystal is shown in Fig. 3.

### Experimental

The compound was obtained commercially (Schuchardt). Crystals suitable for the X-ray diffraction study were taken directly from the provided batch.

### Refinement

Carbon-bound H atoms were placed in calculated positions (C—H 0.99 Å for the methylene group and C—H 0.95 Å for aromatic C atoms) and were included in the refinement in the riding model approximation, with *U*(H) set to 1.2*U*_eq_(C).

### Crystal data

**Table:**

C_8_H_7_BrO	*F*(000) = 392
*M**_r_* = 199.05	*D*_x_ = 1.752 Mg m^−^^3^
Orthorhombic, *P*2_1_2_1_2_1_	Mo *K*α radiation, λ = 0.71073 Å
Hall symbol: P 2ac 2ab	Cell parameters from 5571 reflections
*a* = 4.1459 (2) Å	θ = 2.4–28.2°
*b* = 9.6731 (5) Å	µ = 5.37 mm^−^^1^
*c* = 18.8178 (9) Å	*T* = 200 K
*V* = 754.66 (6) Å^3^	Platelet, colourless
*Z* = 4	0.54 × 0.43 × 0.09 mm

### Data collection

**Table:**

Bruker APEXII CCD diffractometer	1867 independent reflections
Radiation source: fine-focus sealed tube	1692 reflections with *I* > 2σ(*I*)
graphite	*R*_int_ = 0.037
φ and ω scans	θ_max_ = 28.3°, θ_min_ = 3.9°
Absorption correction: multi-scan (*SADABS*; Bruker, 2010)	*h* = −4→5
*T*_min_ = 0.588, *T*_max_ = 1.000	*k* = −12→12
7436 measured reflections	*l* = −25→24

### Refinement

**Table:**

Refinement on *F*^2^	Secondary atom site location: difference Fourier map
Least-squares matrix: full	Hydrogen site location: inferred from neighbouring sites
*R*[*F*^2^ > 2σ(*F*^2^)] = 0.029	H-atom parameters constrained
*wR*(*F*^2^) = 0.072	*w* = 1/[σ^2^(*F*_o_^2^) + (0.0377*P*)^2^ + 0.0956*P*] where *P* = (*F*_o_^2^ + 2*F*_c_^2^)/3
*S* = 1.08	(Δ/σ)_max_ < 0.001
1867 reflections	Δρ_max_ = 0.42 e Å^−^^3^
91 parameters	Δρ_min_ = −0.66 e Å^−^^3^
0 restraints	Absolute structure: Flack (1983), with 736 Friedel pairs
Primary atom site location: structure-invariant direct methods	Flack parameter: 0.015 (14)

### Fractional atomic coordinates and isotropic or equivalent isotropic displacement parameters (Å^2^)

**Table:**

	*x*	*y*	*z*	*U*_iso_*/*U*_eq_	
Br1	0.94179 (9)	0.02569 (3)	0.353315 (15)	0.04357 (12)	
O1	0.7669 (6)	−0.1520 (2)	0.22942 (12)	0.0462 (6)	
C1	0.6825 (7)	−0.0344 (3)	0.21549 (14)	0.0288 (5)	
C2	0.7424 (7)	0.0837 (3)	0.26609 (14)	0.0302 (6)	
H21	0.5345	0.1292	0.2771	0.036*	
H22	0.8828	0.1527	0.2426	0.036*	
C3	0.5113 (6)	−0.0030 (2)	0.14809 (12)	0.0269 (5)	
C4	0.4564 (9)	−0.1109 (3)	0.10021 (14)	0.0361 (6)	
H4	0.5303	−0.2014	0.1111	0.043*	
C5	0.2955 (9)	−0.0866 (3)	0.03729 (16)	0.0416 (7)	
H5	0.2609	−0.1604	0.0049	0.050*	
C6	0.1839 (9)	0.0449 (4)	0.02105 (16)	0.0400 (7)	
H6	0.0712	0.0610	−0.0221	0.048*	
C7	0.2374 (8)	0.1525 (3)	0.06797 (14)	0.0355 (7)	
H7	0.1621	0.2426	0.0569	0.043*	
C8	0.4002 (7)	0.1294 (3)	0.13102 (14)	0.0303 (6)	
H8	0.4366	0.2039	0.1629	0.036*	

### Atomic displacement parameters (Å^2^)

**Table:**

	*U*^11^	*U*^22^	*U*^33^	*U*^12^	*U*^13^	*U*^23^
Br1	0.04576 (18)	0.04907 (17)	0.03589 (16)	−0.00752 (13)	−0.01195 (14)	0.01138 (12)
O1	0.0662 (17)	0.0267 (9)	0.0455 (12)	0.0095 (11)	−0.0047 (13)	0.0036 (8)
C1	0.0309 (13)	0.0263 (11)	0.0293 (13)	0.0013 (11)	0.0051 (11)	0.0042 (10)
C2	0.0328 (15)	0.0308 (12)	0.0270 (12)	−0.0025 (12)	−0.0040 (12)	0.0023 (11)
C3	0.0288 (13)	0.0273 (12)	0.0246 (11)	−0.0022 (8)	0.0046 (12)	0.0001 (9)
C4	0.0474 (17)	0.0269 (11)	0.0341 (14)	−0.0029 (13)	0.0083 (15)	−0.0029 (9)
C5	0.052 (2)	0.0408 (15)	0.0325 (15)	−0.0080 (16)	0.0017 (16)	−0.0121 (13)
C6	0.0385 (16)	0.0567 (19)	0.0247 (13)	−0.0042 (14)	−0.0012 (13)	0.0008 (12)
C7	0.0393 (18)	0.0386 (14)	0.0286 (14)	0.0046 (13)	0.0011 (14)	0.0028 (11)
C8	0.0360 (16)	0.0288 (12)	0.0262 (13)	−0.0008 (11)	0.0026 (12)	−0.0005 (9)

### Geometric parameters (Å, °)

**Table:**

Br1—C2	1.922 (3)	C4—H4	0.9500
O1—C1	1.219 (3)	C5—C6	1.388 (5)
C1—C3	1.485 (4)	C5—H5	0.9500
C1—C2	1.508 (4)	C6—C7	1.382 (4)
C2—H21	0.9900	C6—H6	0.9500
C2—H22	0.9900	C7—C8	1.383 (4)
C3—C4	1.397 (3)	C7—H7	0.9500
C3—C8	1.399 (3)	C8—H8	0.9500
C4—C5	1.379 (4)		
			
O1—C1—C3	120.8 (2)	C3—C4—H4	119.8
O1—C1—C2	121.6 (3)	C4—C5—C6	120.4 (3)
C3—C1—C2	117.6 (2)	C4—C5—H5	119.8
C1—C2—Br1	112.88 (19)	C6—C5—H5	119.8
C1—C2—H21	109.0	C7—C6—C5	119.7 (3)
Br1—C2—H21	109.0	C7—C6—H6	120.1
C1—C2—H22	109.0	C5—C6—H6	120.1
Br1—C2—H22	109.0	C6—C7—C8	120.3 (3)
H21—C2—H22	107.8	C6—C7—H7	119.8
C4—C3—C8	118.8 (2)	C8—C7—H7	119.8
C4—C3—C1	118.4 (2)	C7—C8—C3	120.4 (2)
C8—C3—C1	122.8 (2)	C7—C8—H8	119.8
C5—C4—C3	120.4 (3)	C3—C8—H8	119.8
C5—C4—H4	119.8		
			
O1—C1—C2—Br1	−2.6 (4)	C1—C3—C4—C5	179.4 (3)
C3—C1—C2—Br1	176.40 (19)	C3—C4—C5—C6	−0.5 (5)
O1—C1—C3—C4	−1.6 (4)	C4—C5—C6—C7	0.7 (5)
C2—C1—C3—C4	179.4 (3)	C5—C6—C7—C8	−0.3 (5)
O1—C1—C3—C8	177.7 (3)	C6—C7—C8—C3	−0.2 (4)
C2—C1—C3—C8	−1.3 (4)	C4—C3—C8—C7	0.3 (4)
C8—C3—C4—C5	0.0 (4)	C1—C3—C8—C7	−179.0 (3)

### Hydrogen-bond geometry (Å, °)

**Table:**

*D*—H···*A*	*D*—H	H···*A*	*D*···*A*	*D*—H···*A*
C2—H21···O1^i^	0.99	2.46	3.317 (4)	145
C2—H22···O1^ii^	0.99	2.44	3.268 (4)	141
C8—H8···O1^i^	0.95	2.60	3.442 (3)	148

Symmetry codes: (i) −*x*+1, *y*+1/2, −*z*+1/2; (ii) −*x*+2, *y*+1/2, −*z*+1/2.
